# Neuroimaging in Vascular Parkinsonism

**DOI:** 10.1007/s11910-019-1019-7

**Published:** 2019-11-26

**Authors:** Karen K. Y. Ma, Shi Lin, Vincent C. T. Mok

**Affiliations:** 1 Division of Neurology, Department of Medicine and Therapeutics, Prince of Wales Hospital, Faculty of Medicine, The Chinese University of Hong Kong, Hong Kong SAR, China; 2 Margaret K.L. Cheung Research Centre for Management of Parkinsonism, Gerald Choa Neuroscience Centre, Lui Che Woo Institute of Innovative Medicine, The Chinese University of Hong Kong, Hong Kong SAR, China; 3 Department of Imaging & Interventional Radiology, Prince of Wales Hospital, Faculty of Medicine, The Chinese University of Hong Kong, Hong Kong SAR, China; 4 BrainNow Research Institute, Guangdong Province, Shenzhen, China

**Keywords:** Vascular parkinsonism, Atypical parkinsonism, Magnetic resonance imaging volumetry, Diffusion tensor imaging, ^123^I-FP-CIT dopamine transporter imaging

## Abstract

**Purpose of Review:**

Being a disease with heterogeneous presentations and unclear consensus on its diagnostic criteria, it is difficult to differentiate vascular parkinsonism (VaP) from other neurodegenerative parkinsonism variants. Ongoing research on structural and functional neuroimaging targeting dopaminergic pathway provides us more insight into the pathophysiology of VaP to improve diagnostic accuracy. The aim of this article is to review how the emerging imaging modalities help the diagnostic process and treatment decision in VaP.

**Recent Findings:**

Dopamine transporter imaging is a promising tool in differentiating presynaptic parkinsonism and VaP. It also predicts the levodopa responders in VaP. Advanced MRI techniques including volumetry, diffusion tensor imaging and sequences visualising substantia nigra are under development, and they are complementary to each other in detecting structural and functional changes in VaP, which is crucial to ensure the quality of future therapeutic trials for VaP.

**Summary:**

Dopamine transporter imaging is recommended to patients with suspected VaP. Multimodal MRI in VaP would be an important area to be investigated in the near future.

## Introduction

The key features of parkinsonism are rigidity, tremor, bradykinesia and postural instability. Idiopathic Parkinson’s disease (IPD) is the most common cause of neurodegenerative parkinsonism. Atypical parkinsonism including multiple system atrophy (MSA), progressive supranuclear palsy (PSP), corticobasal degeneration (CBD) and dementia of Lewy body (DLB) contribute to the vast majority of the remaining neurodegenerative parkinsonian syndromes [1]. The pathophysiology of neurodegenerative parkinsonian syndromes was proposed to be the result of the degeneration of dopaminergic neurons in the nigrostriatal pathway due to abnormal alpha-synuclein or tau deposition [2, 3]. In patients with IPD, resting tremor, unilateral onset of symptoms, dramatic response to dopamine replacement therapies and L-dopa related dyskinesia are the characteristic features [4]. Among all atypical parkinsonian syndromes, IPD, PSP and MSA are the most difficult to be differentiated due to their similar signs and symptoms in early stage of disease and sometimes late presentation of the signature signs, such as cerebellar ataxia or autonomic dysfunction in MSA, vertical gaze palsy and postural instability with recurrent fall in PSP [3, 5]. The latter two atypical parkinsonism syndromes progress rapidly and relentlessly when compared with IPD [6]. The median time from disease onset to death is around 6 to 9 years in PSP and 5 to 10 years in MSA [7–10].

On the other hand, parkinsonism can also result from vascular insult, hypoxia, drug, infection or trauma, which are grouped as secondary parkinsonism [11–14]. In this article, we would like to focus on vascular parkinsonism (VaP). It contributes to 3–5% of patients with parkinsonism from a post-mortem study [15]. VaP represents a heterogeneous group of parkinsonism syndromes caused by small vessel diseases. In 1929, Critchley first described the term ‘atherosclerotic parkinsonism’, which was of rapid onset with the absence of tremor, muscles were firmer to touch. It was associated with vascular changes more in the globus pallidus, to a lesser extent in the substantia nigra, and usually happened in hypertensive individuals [16]. Epidemiological studies in later decades also echoed the above observations in VaP [17]. Additional features of VaP include onset to be 4 to10 years later than IPD, predominant lower body involvement causing postural instability and gait difficulty [18, 19]. In VaP, only 20 to 38% patients responded to levodopa compared with 75 to 100% in IPD patients, and motor fluctuation was rarely observed in VaP [20]. Cognitive decline, pseudobulbar palsy and incontinence were more often found in VaP patients in contrast to IPD patients. Pathological studies proposed the mechanism of VaP was probably due to the disruption of cortical basal ganglion connections by vascular insults, causing impairment of cortico-striato-pallido-thalamo-cortical motor loop [21].

Updated VaP subtypes definition includes 3 groups of cases: (1) acute or subacute focal infarct involving striatal dopaminergic system, (2) insidious onset of parkinsonism predominantly affecting gait and postural instability, which is the most frequent VaP subtype, and (3) mixed and overlapping syndrome of neurodegenerative parkinsonism and comorbid vascular lesions which increase the parkinsonism impairment [22••]. To complicate further, some of the so-called VaP were actually vascular pseudoparkinsonism with gait instability and apathetic depression caused by frontal or subcortical strokes, while some were pseudovascular pseudoparkinsonism with higher level gait disorder caused by condition such as normal pressure hydrocephalus [23].

The presence of white matter lesion in patients with parkinsonism does not necessarily imply the diagnosis of VaP [24]. White matter lesions might be present in IPD and other atypical parkinsonian syndromes. Cerebral vascular lesions were found in 19–50% of pathologically confirmed IPD patients in various studies [15, 25, 26]. The exact role of small vessel disease in parkinsonism is uncertain. Some postulated small vessel disease lowered the threshold for Lewy body disease to manifest its parkinsonism symptoms [27]. A study showed that higher white matter hyperintensity (WMH) volume and larger lacunar infarct numbers increased the 5 years risk of any parkinsonism with hazard ratio 1.8 per standard deviation (SD) increase and 1.4 per number increase respectively [28]. White matter lesion burden was also associated with lower cognitive score and increase risk in postural instability and gait disturbance in IPD [29].

It is difficult to diagnose VaP due to its clinical heterogeneity and overlapping features with IPD and other atypical parkinsonisms. In an autopsy collection study by Horvath of 261 patients who died with parkinsonism, 23 were confirmed to be VaP pathologically, with only 3 correctly diagnosed to have VaP during their lifetime [30]. In another study from Queen Square brain bank, only 6 out of 28 VaP patients were correctly diagnosed with VaP during their lifetime and the remainder were labelled as IPD or atypical parkinsonism [31]. Meanwhile, 7% of clinical IPD patients had autopsy diagnosis of VaP, and 30–55% of IPD patients with white matter lesions were being incorrectly labelled as VaP [28, 30]. The accuracy of clinical diagnosis of VaP when compared with autopsy findings ranged from 0 to 66% in few clinicopathological studies [32–34]. These results demonstrate the difficulty in diagnosing VaP due to its similar presentations with other parkinsonian syndromes. It is even more difficult to distinguish VaP from atypical parkinsonism, as they share the features of poor response to levodopa and relative symmetrical onset. Among all atypical parkinsonism, it is most difficult to differentiate VaP from PSP. Vascular lesion in the brainstem, thalamus and higher subcortical region in VaP mimics the clinical signs produced by neurodegeneration from midbrain origin in PSP. Pseudobulbar palsy and lower body parkinsonism are common in both VaP and PSP, and some VaP patients also have vertical gaze palsy [ 35–37]. Another important reason of low diagnostic accuracy of VaP is due to poor correlation of vascular lesion with parkinsonism and the coexistence of small vessel disease in neurodegenerative parkinsonian syndromes [29, 38]. It is important to make accurate diagnosis due to the differences in prognosis, treatment response to L-dopa and deep brain stimulation in IPD, VaP and atypical parkinsonism [39, 40]. In addition, proper diagnosis labelling is a prerequisite for recruitment in clinical trials of therapeutics agents for these parkinsonism syndromes.

With the emergence of advanced imaging techniques, diagnosis of pure VaP, mixed VaP with IPD or other neurodegenerative parkinsonism (both IPD and atypical parkinsonism) with coincidental small vessel disease could achieve higher accuracy. Traditionally, we rely on structural imaging by MRI with conventional T1, T2, FLAIR, DWI and ADC, gradient echo sequence or susceptibility weighed images (SWI) showing WMH, lacunar infarct and microbleeds to diagnose VaP [24, 41]. Nowadays, more advanced MRI techniques enables brain volumetry, white matter fibre tractography and visualisation of minute regional atrophy, microinfarcts, functional connectivity and nigrostriatal system activity, which aid the diagnosis and elucidation of the mechanisms of VaP and various neurodegenerative parkinsonian syndromes [42, 43•, 44•, 45•, 46–48]. ^123^FP-CIT SPECT (DaT SCAN), a dopamine transporter imaging technique, can also be used to distinguish VaP from IPD and to determine the responsiveness of levodopa in VaP patients [49–51].

## Magnetic Resonance Imaging

By definition, the presence of vascular lesion would be necessary for the diagnosis of VaP. Conventional MRI is widely used as an adjunctive investigation in the work up of VaP as it is non-invasive and highly sensitive to vascular insult. 90–100% of VaP patients had abnormal MRI compared with 12–23% in IPD [20]. Nearly 67% of IPD patients had non-specific periventricular WMH and around 30% had mild-to-moderate cortical atrophy on brain MRI [19, 52]. In classical VaP cases with lower body parkinsonism, MRI showed periventricular WMH, lacunar infarcts predominantly in the basal ganglion, and dilatation of the lateral and third ventricles [19, 47]. In acute or subacute VaP, MRI brain showed strategic infarcts in the territory of the striatal system [53–55]. A few morphological MRI studies suggested that the presence of microbleeds and subcortical or cortical atrophy were supportive features for VaP [41, 47]. These MRI features were also helpful to prognosticate the risk of acquiring VaP. For example, the presence of microbleeds and low gray matter volume increase the risk of development of VaP with a hazard ratio of 5.7 and 0.4 per SD increase, respectively [28]. Nevertheless, many individuals do not develop parkinsonism despite the presence of white matter lesion [29, 46]. Therefore, it is difficult to distinguish VaP from neurodegenerative parkinsonism with comorbid small vessel disease by conventional MRI. To overcome this limitation, multiple new MRI techniques are currently under investigation in diagnosing VaP, including MRI volumetry, DTI and neuromelanin sensitive sequence (see Table 1).

**Table 1:** MRI characteristics in vascular parkinsonism and neurodegenerative parkinsonism

MRI	VaP	IPD	PSP	MSA
Conventional MRI	Periventricular white matter lesion, lacunar infarcts in the basal ganglion, dilatation of lateral and third ventricles [19,47] Presence of microbleed, subcortical or cortical atrophy [41,47]		Hummingbird sign Morning glory sign	Hot cross bun sign
MRI volumetrics	↑ WMH volume in VaP vs IPD with cutoff of > 0.6% of brain tissue volume [47] ↑ WMH volume and caudate volume in VaP vs IPD and HC [43•] ↓ midbrain area, pons area, midbrain to pons in VaP vs IPD and PSP [25, 44•, 45•] ↓ SNpc width in IPD vs VaP [103]	↓ caudate and putamen volume in IPD vs HC	↓ midbrain area, midbrain to pons ratio, pons area in PSP vs VaP [44•, 45•] ↑ MRPI in PSP vs VaP, MSA, IPD and HC [44•, 45•, 64, 67, 68] ↓ midbrain and SCP volume in PSP vs IPD, MSA and HC [61, 62, 83] ↓ midbrain to pons ratio in PSP vs IPD and MSA [65]	↓ putamen, cerebellum and pons volume in MSA vs IPD and HC [58–60]
Diffusion tensor imaging (DTI)	↓ FA and ↑ MD in frontal lobe, internal capsule, corpus callosum in VaP vs IPD and HC [48, 77, 78•]	↓ FA in substantia nigra in IPD vs HC [74]	↓ FA and ↑ MD in frontal area, midbrain, basal ganglion, SCP in PSP vs IPD [74, 76]	↓ FA and ↑ MD in pons, putamen, MCP in MSA vs IPD [74, 75]
Neuromelanin sensitive MRI		↓ contrast to noise ratio and neuromelanin positive SN volume in IPD vs HC [86, 87]	↓ neuromelanin positive SN volume in PSP vs HC [86]	↓ contrast to noise ratio and neuromelanin positive SN volume in MSA vs HC [86, 87]
Susceptibility weighted imaging (SWI)	Intact dorsal nigral hyperdensity in VaP vs IPD, MSA, PSP [83]	Loss of dorsal nigral hyperdensity in IPD vs HC [84•]	Loss of dorsal nigral hyperdensity in PSP vs HC [84•]	Loss of dorsal nigral hyperdensity in MSA vs HC [84•]

*↓*, decrease; *↑*, increase; *VaP*, vascular parkinsonism; *IPD*, idiopathic Parkinson’s disease; *PSP*, progressive supranuclear palsy; *HC*, healthy control; *MSA*, multiple system atrophy; *WMHL*, white matter hyperdense lesion; *MRPI*, magnetic resonance parkinsonism index; *M/P*, midbrain to pons ratio; *SCP*, superior cerebellar peduncle; *MCP*, middle cerebellar peduncle; *SNpc*, substantia nigra pars compacta; *SN*, substantia nigra; *FA*, fractional anisotropy; *MD*, mean diffusivity

### MRI Volumetry

MRI volumetry comprises acquisition of 3D T1-weighted sequences which allows quantification of regional brain volumes using different post-processing software and some are fully automated. White matter hyperintensity volume and load, size of subcortical structures and measure of the volumes of various regions of interest (ROIs) are measured using this technique. It is well studied in IPD and atypical parkinsonism. In IPD, the volumes of caudate and putamen were smaller compared with healthy control [56, 57]. In MSA, putamen, cerebellum and pons volumes were smaller compared with IPD and healthy control [58–60]. Volumes of the midbrain and superior cerebellar peduncle (SCP) were smaller in PSP when compared with IPD, MSA and healthy control [61–64].The midbrain to pons ratio was found to be significantly smaller in PSP when compared with IPD, MSA and healthy control and could serve as a diagnostic marker with high accuracy [65]. Similarly, a ratio named magnetic resonance parkinsonism index (MRPI), which was calculated by multiplying the pons to midbrain area ratio (P/M) by the middle cerebellar peduncle (MCP) width to SCP width ratio (MCP/SCP), was significantly higher in PSP, and with 100% sensitivity and specificity to distinguish PSP from IPD, MSA-P and control [66]. These results were reproducible in different studies [65, 67, 68]. Midbrain to pons ratio would be a more practical and convenient method than MRPI which can be measured in a single slice on midsagittal plane [65].

In VaP, MRI volumetry can be employed to calculate volumes of WMH and brainstem structures. WMH volume was reported to be larger and brainstem volume smaller in VaP when compared with neurodegenerative parkinsonism. Various visual rating scores of white matter lesion, such as Fazekas scale, were used to estimate the ischemia load which correlated with disease severity in terms of motor and activity of daily living score [69–71]. However, these scores cannot provide the details of the locations of the lesion and are unable to give the absolute volume. Two decades ago, a volumetric study by manual stereologic estimation suggested that subcortical white or gray matter lesion volume was significantly higher in VaP than that in IPD, with a cutoff point of 0.6% of subcortical white or gray matter lesion to brain tissue volume was best to discriminate VaP from IPD [47]. Nonetheless, it is time consuming to perform manual volume calculation and it was subjected to interrater discrepancy. With the recent advancement in automated volumetric analysis, both WMH quantitation using FLAIR MRI and regional brain volumetry using 3D T1 can be facilitated by a single automated volumetry method with precision and a short amount of time. Besides, subtle differences in regional brain volumes between VaP and neurodegenerative parkinsonism can be accurately measured by automated volumetric analysis and a few promising diagnostic markers have emerged. In an automated volumetric morphometry study, VaP patients had higher WMH volume than control (*p* = 0.0002) and IPD (*p* = 0.011) patients, as well as higher caudate nucleus volume than control (*p* = 0.011) and IPD (*p* = 0.038). WMH volume, a marker of white matter ischemia severity, was found to be positively correlated with caudate volume (*p* < 0.0001) [43•] and was consistent with a previous finding of increased ipsilateral caudate gray matter volume after striatal cortical stroke [72]. It was postulated that vascular events would cause remodeling and neuronal reorganisation of the cortico-striato-thalamic loop, hence causing caudate hypertrophy which can be a potential marker in VaP.

Brainstem structures atrophy were proven to be less prominent in IPD than other neurodegenerative parkinsonism in multiple reports. VaP patients had significantly smaller midbrain area compared with IPD [42], which might account for the features of early postural instability, pseudobulbar symptoms and lower body dominance in VaP. However, the cause of midbrain atrophy in VaP is not well understood. It might be related to the pathophysiology of VaP or as a result of secondary axonal degeneration following supratentorial cortical atrophy. Caution has to be taken in the interpretation of the midbrain area which might not directly correlate with midbrain volume. MRI volumetry studies also confirmed midbrain atrophy was a characteristic pathognomonic feature that distinguishes PSP from other parkinsonian syndromes [61–64, 73], which were consistent with the classical hummingbird sign caused by midbrain atrophy in PSP. Indeed, the clinical presentations in PSP and VaP are similar which can cause diagnostic difficulty. Degree of midbrain atrophy by midbrain area was significantly lower in PSP compared with VaP, so as the superior cerebellar peduncle volume and midbrain to pons ratio [45•]. By the same token, MRPI is not only useful to distinguish PSP from IPD and MSA, but VaP as well. MRPI was significantly lower in PSP when compared with VaP with the value of > 13 having 100% sensitivity and specificity to differentiate these two conditions with a very high accuracy suggesting it was a reliable marker. However, there was no significant difference in MRPI values between IPD and VaP [44•].

Accumulating evidence suggesting a role of MRI volumetry to distinguish VaP from other neurodegenerative parkinsonism. The number of evidence of MRI volumetrics in VaP was quite substantial. The advantage of MRI volumetry is that it is an objective investigation to quantify the white matter lesions and atrophy of specific ROI in a short period of time. On the other hand, the post-processing softwares were not unified and might not be consistent with each other and there was no standardized validation available both technically and clinically. Large scale study would be needed to validate the reliability of different volumetrics calculation methods. Besides, serial scans would be helpful to compensate for the limitation of cross-sectional MRI studies.

### Diffusion Tensor Imaging

Diffusion tensor imaging (DTI) permits studies on the effect of vascular lesion on anatomically well-defined fibre tracts. DTI measures the direction and the magnitude of water diffusion in brain tissues quantitatively, therefore reflecting the integrity and orientation of white matter tracts by measuring the fractional anisotropy (FA) and mean diffusivity (MD). Lower FA and higher MD values suggest increasing disruption of white matter tracts and water content, respectively. FA in substantia nigra was shown to be reduced in IPD compared with healthy control, while FA was reduced and MD was increased in pons, cerebellum, MCP and putamen in MSA when compared with IPD and healthy controls, and FA was reduced and MD was increased in frontal white matter, midbrain, basal ganglion and SCP in PSP when compared with IPD [74–76]. DTI is an emerging research tool to differentiate IPD and other neurodegenerative parkinsonism. Because of the unique property of DTI in detecting white matter tract integrity by the flow of free water molecules, it has gained increased interest in the diagnostic process of VaP and understanding of the pathophysiology of VaP. Tract based spatial statistics (TBSS) analysis were performed in VaP patients by various groups. One study correlates the disease severity in terms of Unified Parkinson’s Disease Rating Scale scores and modified postural instability gait difficulty (PIGD) scores in VaP and healthy control. The fronto-thalamo-capsular region showed significantly lower regional fractional anisotropy in VaP compared with the control group. The mean FA of fibre tracts from the bilateral frontal lobe to anterior limb of the internal capsule and genu of the corpus callosum was negatively correlating to these scores. It suggested that frontal white matter microstructural disconnection contributes to the clinical features in VaP [48].

Other studies showed that there was a significant reduction in FA and an increase in MD in normal-appearing white matter in corpus callosum, internal and external capsule and corona radiata in VaP than IPD and controls. WMH volume and location were found to be significantly related to the damage of normal-appearing white matter in DTI [77]. DTI was able to detect white matter disconnectivity in VaP before structural lesions become noticeable and can assist earlier diagnosis of VaP [78•]. DTI is sensitive in detecting white matter lesion, but it is not possible to differentiate whether the parkinsonism features are due the vascular lesion or concomitant neurodegenerative parkinsonism. Moreover, the results on the use of DTI in parkinsonian syndromes and VaP were limited and exploratory with no conclusion available on its diagnostic accuracy currently.

### Iron Sensitive and Neuromelanin Sensitive MRI

Dorsal nigral hyperintensity abnormalities can now be visualised by the recent development of MRI techniques. Within the substantia nigra, it contains iron and neuromelanin granules. Iron accumulation and neuromelanin loss are found to be associated with dopaminergic degeneration in the substantia nigra [79]. The iron content in the substantia nigra was found to be 30% higher in IPD than healthy controls in pathological studies [80]. The amount of neuromelanin was reduced when PD progressed and was confirmed in a number of post-mortem studies [81–83].

T2* and SWI sequences in 3Tesla or 7Tesla are sensitive to detect the iron load in the dorsolateral substantia nigra and these signals are inversely proportional to the iron content. Meta-analyses showed that in iron sensitive MRI sequences, there was loss of dorsal nigral hyperintensity in IPD and atypical parkinsonism due to the increased iron deposition [84•]. These techniques were used to differentiate nigrostriatal parkinsonism from healthy controls, but it cannot differentiate between IPD and atypical parkinsonism. The number of VaP patients included in substantia nigra imaging was small. In a study, 14 out of 19 VaP patients had intact dorsal nigral hyperintensity, which substantiated the observation that majority of VaP were not caused by substantia nigra degeneration [85].

Neuromelanin has paramagnetic properties and T1 shortening effect when combining with metal including copper and iron. In T1-weighted fast spin-echo sequence of MRI, neuromelanin containing nuclei would be visualised as distinct hyperintensity when compared with other structures. Neuromelanin related signals in the brainstem area and substantia nigra volume were significantly reduced in IPD, PSP, MSA and CBD patients when compared with healthy control [86, 87]. In essential tremor, the area and width of the substantia nigra in neuromelanin sensitive sequence were significantly higher than IPD [88]. Up to date, there is no study available on the utility of neuromelanin sensitive sequence in VaP.

The nature of this nigrosome imaging partially resembles DaTScan in evaluating the loss and dysfunction of nigrostriatal dopaminergic neurons. Large portion of parkinsonism patients with loss of dorsal nigral hyperintensity in SWI were having dopaminergic degeneration as indicated by DaTScan [85]. The relationship between neuromelanin-sensitive MRI and dopamine loss in a striatal system measured by ^123^I-FP-CIT SPECT was being investigated. Both substantia nigra volume and contrast to noise ratio in neuromelanin sensitive sequence was shown to have a positive correlation with the striatal uptake as reflected by the specific binding ratio of ^123^I-FP-CIT in IPD patients. The asymmetry index of neuromelanin positive substantia nigra volume was also positively correlating with the asymmetry index of the specific binding ratio of ^123^I-FP-CIT in DaTScan [89]. The asymmetry index of bilateral striatal ^123^I-FP-CIT uptake is a marker to differentiate IPD and VaP, which will be further elaborated later in this article [49–51]. Based on these findings, we can extrapolate that the asymmetry index of neuromelanin positive substantia nigra volume may be a possible marker to distinguish IPD and VaP. Further studies in the future are needed to explore the application of nigrosome imaging in VaP.

The quality of the SWI sequence is prone to be affected by motion artefact and it has great inter-observer variability. For neuromelanin sensitive MRI, the imaging protocol and method analysis are not yet standardized. Larger studies involving multiple centres would be helpful to overcome this limitation.

## Molecular Imaging

Molecular imaging such as SPECT (single positive emission computed tomography) and PET (positive emission tomography) are techniques that help diagnosis of parkinsonian syndromes by assessing the neurotransmitter activities, especially the dopaminergic transporters [90–92] (see Table 2).

**Table 2:** Molecular imaging findings in vascular parkinsonism

Molecular imaging	Findings
^123^FP-CIT SPECT (DaTScan)	- Normal scan in 32.5% of VaP patients and abnormal in all IPD patients [49] - ↑ uptake ratio for most affected putamen and ipsilateral caudate and striatum in VaP vs IPD [49, 50] - ↓ Striatal asymmetry index in VaP vs IPD [49–51, 97] - “Punched out” uptake and reduced uptake at area congruous to focal infarct in VaP [49] - Normal scan suggests negative response to levodopa treatment [49, 96]
PET	
FDG and F DOPA	- ↓ F-Dopa update (putamen > caudate), ↓ frontal and cerebellum FDG uptake and ↑ thalamus FDG uptake in a case of sudden onset right sided parkinsonism and bilateral (left > right) SN lesion [100]
^18^F-FP-CIT	- ↓ Left putamen uptake in a case of right sided parkinsonism and left midbrain infarct on MRI [102] - ↓ Left caudate head and anterior putamen uptake in a case of right sided parkinsonism with corresponding old infarct on MRI [102] - ↓ Right thalamic uptake in a case of left sided parkinsonism and corresponding old infarct in MRI [102] - ↓ Left caudate and putamen uptake in a case of right side predominant parkinsonism and left midbrain infarct [101]

↓, decrease; ↑, increase; *VaP*, vascular parkinsonism; *IPD*, idiopathic Parkinson’s disease; *PET*, positive emission tomography; *FDG*, 18-F-flurodeoxyglucose; *SN*, substantia nigra

### Dopamine Transporter Single Photon Emission Computerised Tomography (DaTScan)

^123^FP-CIT SPECT (DaTScan) is one of the most well-established molecular imaging technique to differentiate presynaptic parkinsonism from other parkinsonism such as VaP, drug-induced parkinsonism and psychogenic disorder [93]. It uses ^123^FP-CIT, which is a cocaine derivative, as dopamine transporter ligand, and it is highly sensitive to detect nigrostriatal pathway degeneration. Other cocaine derivatives ligands, for example, ^123^b-CIT and 99m-Tc TRODAT, were used to serve the same purpose in some individual studies. In IPD and atypical parkinsonism, there was marked reduction in striatal uptake with putamen being the most affected in IPD [90]. The degree of reduction in ^123^FP-CIT striatal binding correlated with the clinical motor severity in IPD [94]. In VaP, the patterns of ^123^FP-CIT uptake are variable due to the heterogeneity of its clinical subtypes. DaTScan was found to be normal in 32.5% of VaP patients and abnormal in all IPD patients described by Benitez-Rivero et al. [49] Many studies showed the degree of ^123^FP-CIT striatal uptake in VaP were preserved or mildly reduced only. Some of the VaP patients were reported to have a reduction in striatal uptake as marked as IPD patients [49–51]. In some acute or subacute VaP cases, a significant reduction in ^123^FP-CIT striatal uptake ipsilateral to the infarcts was observed. The exact reason for this observation is still unknown. Some groups assumed that it was because of the overlapping presynaptic parkinsonism with comorbid VaP or coincidence of small vessel disease in those patients with abnormal DaTScan. Another hypothesis was cerebral vascular insult might induce asymmetrical striatal dopaminergic degeneration [95]. Other than understanding the pathophysiology of VaP, DaTScan could predict response to levodopa in VaP patients. A study showed that 93% VaP patients with normal DaTScan and 48% VaP patients with abnormal DaTScan had a negative response to levodopa [96]. DaTScan can select the group of VaP patients with the presence of nigrostriatal dopaminergic degeneration, who are more likely to response to levodopa. The level of treatment response represented by the percentage change of UPDRS after levodopa was not correlated with the reduction in striatal ^123^FP-CIT uptakes reported by Zijlman et al., but this could be limited by the small number of VaP patients in this study [51]. It is recommended to perform DaTScan in clinical VaP patients for better treatment selection.

In IPD and other atypical parkinsonism, DaTScan cannot be used to differentiate them. On the contrary, DaTScan was useful in distinguish VaP and IPD. Comparing IPD and VaP, the asymmetry index between the right to left ^123^FP-CIT striatal uptake was similar in VaP but higher in IPD, and the caudate and putamen uptake and the putamen to caudate ratio of the most affected side were higher in VaP than IPD both visually and semi-quantitatively [51].The result was reproducible by different studies [49, 50, 97]. There was no significant difference in the asymmetry index of right to left striatum between VaP and healthy controls. These findings are consistent with the diffuse nature of the disease process underlying VaP versus persistent asymmetric nigrostriatal degeneration in IPD. The asymmetry index between bilateral striatal tracer uptake was a promising marker to differentiate VaP from IPD. In a meta-analysis, DaTScan was found to have a sensitivity of 80–100% and specificity of 73–100% to differentiate IPD and VaP [98]. In summary, DaTScan would be a useful ancillary test in VaP that could improve the diagnostic accuracy and prognosticate patient’s response to levodopa treatment. If resources allowed, suspected VaP patients should undergo DaTScan.

### Positive Emission Tomography

Positive emission tomography (PET) is another metabolic imaging used for the investigation of parkinsonian syndromes. It provides better spatial resolution than SPECT. PET can assess the functional abnormalities in the early stage of various neurodegenerative parkinsonian syndromes before structural changes appeared in a relatively late stage in the disease [91, 92]. It is divided into quantification of regional glucose metabolism or dopaminergic pathway metabolism at the presynaptic or synaptic level by using different substrates. 18-F-flurodeoxyglucose positron emission tomography (FDG-PET) assesses the resting cerebral glucose metabolism to differentiate IPD and various atypical parkinsonism by identifying the specific pattern of metabolism in each syndrome. Concordance of FDG-PET and clinical diagnosis was 92% (IPD 93%, MSA 90%, PSP 91%, CBS 100%) in overall samples with a total of 70 patients with various parkinsonian syndromes in a recent trial in 2017. The diagnostic accuracy of FDG-PET was 93% for IPD and MSA and 97% for PSP, in contrast to 80% in IPD and 25–50% in MSA by clinical assessment [99]. ^18^F-DOPA is another substrate used to differentiate presynaptic and non-presynaptic parkinsonism. Scattered case reports were available on PET in unilateral parkinsonism after strategic ischemic infarct. A study showed acute VaP caused by focal white matter ischemia in substantia nigra had higher reduction in F-DOPA uptake in putamen than caudate, and hypometabolism at frontal cortex and hypermetabolism at lentiform nucleus and thalamus in FDG-PET, which were similar to IPD [100]. Few case reports illustrated the use of [18F] N-(3-fluoropropyl)-2b-carbon ethoxy-3b-(4-iodophenyl) nortropane (FP-CIT) as dopamine transporter substrate in dual-phase PET in parkinsonism caused by focal infarct. The results were variable with one case showed normal striatal uptake and two cases showed a reduction in uptake at ipsilateral striatum in the early phase and late phase [101, 102]. For insidious onset VaP, there was no PET imaging study so far. More research on the utilisation of PET in VaP is needed to determine its role in clinical use.

## Conclusion

Neuroimaging is essential in diagnosis and understanding of the pathogenesis of VaP. The presence of small vessel disease supports the diagnosis of VaP, but it cannot exclude the existence of other nigrostriatal degeneration parkinsonism. Based on DTI, the disruption of white matter in VaP started from bilateral frontal white matter to striatocapsular fibres. In VaP, caudate hypertrophy and brainstem atrophy predominantly in midbrain were detected by MRI volumetrics study. These results can possibly be secondary to the axonal injury caused by vascular insults. At the same time, MRI volumetry can help distinguish VaP from IPD and PSP. DaTScan can differentiate VaP from presynaptic parkinsonism and guide the decision of starting levodopa in VaP. It is difficult to distinguish the patterns of metabolism by different substrates in PET between VaP and IPD.

Because of the heterogeneity of VaP and the lack of consensus in its diagnostic criteria, the labelling of VaP in existing neuroimaging studies was not congruent and the accuracy of the study results was affected. The sample size of each individual study was small and the imaging protocols were highly variable, causing difficulty to compare or integrate the findings of each study. Moreover, most of these studies were based on clinical and radiological manifestation only without confirmation by autopsy. More post-mortem studies are necessary to validate the clinical diagnosis. Hopefully, with more systemic and robust diagnostic criteria, the results of neuroimaging studies in VaP would be more precise and harmonised. At the moment, except for a trial of levodopa, there is no effective symptomatic treatment for VaP and we can only resort to vascular risk factor control in the hope to slow down the disease process. Neuroimaging of high diagnostic accuracy would be important for correct disease labelling for recruiting subjects into future clinical trials evaluating putative therapeutic agents for VaP.
